# Loin to groin pain –A case report of an intermittent obturator hernia mimicking ureteric colic

**DOI:** 10.1016/j.ijscr.2019.12.025

**Published:** 2019-12-28

**Authors:** Matheesha Herath, Harsh Kanhere

**Affiliations:** a Port Augusta Hospital, 71 Hospital Road Port Augusta, South Australia, 5700, Australia; b The Royal Adelaide Hospital, Port Road, Adelaide, South Australia, 5000, Australia; c The Queen Elizabeth Hospital, Woodville Road, Woodville, South Australia, Australia

**Keywords:** Obturator hernia, Emergency surgery, Laparoscopic surgery, Diagnostic dilemma, Case report, Hernia

## Abstract

**Introduction:**

Obturator hernia is a rare condition and can cause significant diagnostic challenges due to a lack of reliable clinical examination signs. Presentations can have a variety of features and it is a diagnosis that needs to be considered – especially in elderly multiparous women.

**Presentation of case:**

We present a 76-year-old female who has multiple presentations to the Emergency Department (ED) with transient episodes of severe loin to groin pain. Imaging Computer Tomography (CT) initially demonstrated a mild left hydronephrosis and she underwent an unremarkable ureteroscopy and stenting. Following stent removal she continued to have recurrent episodes of the pain. She presented to the ED with one such episode. A repeat CT scan was performed and this demonstrated an obturator hernia with partial small bowel obstruction. She underwent a laparoscopy by which time the hernia had reduced and her pain had settled. Laparoscopy revealed bilateral obturator herniae with the one on left larger than the right. Both were repaired laparoscopically and she made an uneventful recovery.

**Discussion:**

Obturator hernia is an uncommon condition and can have a varied presentation. Comprehensive review of literature demonstrates the difficulty in making accurate diagnosis. Open intervention was the initial gold standard of treatment but there is a growing body of evidence advocating for the use of laparoscopy in an emergency setting.

**Conclusion:**

Early diagnostic imaging with CT while a patient is symptomatic can aid in making an accurate diagnosis. Laparoscopic repair can be safely used with good outcome in the context of an incarcerated obturator hernia.

## Highlights

• Obturator hernia causes significant diagnostic dilemma due to a lack of clinical signs
• We present a case report of an elderly female who presents with atypical transient symptoms mimicking ureteric colic.
• Timing of imaging studies with patient symptoms proved vital to establishing a diagnosis.
• A laparoscopic repair was safely conducted with good results.

## 1 Introduction

Obturator hernia is a very rare surgical pathology representing between 0.05% and 1.4% of all herniae. [[1], [2], [3], [4], [5], [6]] It typically occurs in thin, multiparous, elderly females and is suspected to be due to laxity of pelvic floor muscles [1,5,[7], [8], [9]]. The diagnosis of an obturator hernia can be challenging due to lack of clinical signs [10]. The Howship-Romberg sign, described as an exacerbation of medial thigh pain following hip flexion and external rotation, has been reported present in patients with obturator hernia in 30–67 % of cases [5,11,12]. This sign is often falsely positive in patients who have osteoarthritis, a disease often occurring concurrently within the population who have obturator herniae [5,8]. Given the unreliability of examination findings, diagnosis is typically made with Computer Tomography (CT) imaging [3,13].

The obturator foramen is formed by the pubic bones and the ischial rami. The majority of the foramen is occupied by a membrane with small orifice at the caudal aspect to allow passage of the obturator vein, artery and nerve. [14] Incarceration of peritoneal contents -usually small intestine- within this canal causes discomfort and often obstruction. The associated sequelae of strangulating viscera compounded by diagnostic delay contributes to a high mortality rate of 12 %–70%. (2, 4, 8, 15, 16)

The following case is an unheard-of presentation of an exceptionally rare surgical issue.

This work has been reported in line with SCARE criteria. [17]

## 2 Presentation of case

Our patient is a 76 year old multiparous female who is very fit and independent. Her Body Mass Index is 24 and she had no significant background history. She experienced 5 attacks of debilitating colicky left flank pain radiating to her groin over a period of 5 months. Each attack lasted several hours and resolved after administration of analgesia. During the episodes the patient was passing flatus, not vomiting and there were no precipitants for her symptoms. Whilst in pain her she had a soft abdomen with lower abdominal and left renal angle tenderness. PV examination performed during her second presentation was normal. Howship – Romberg sign was not checked as the patient had osteoarthritis. She underwent several CT scans to investigate her symptoms – all of which were unremarkable aside from mild left sided hydronephrosis, however; her pain had always resolved by the time of imaging. She underwent elective ureteroscopy and stenting to investigate these symptoms which was unremarkable. During her 5^th^ presentation to hospital with identical symptoms CT imaging demonstrated an incarcerated left obturator hernia causing a partial bowel obstruction (Fig. 2, Fig. 33 and Fig. 44). Within an hour following admission the patients’ pain had completely resolved again. Given the CT findings she was booked for a laparoscopy and repair as a semi- urgent procedure.

A Trans Abdominal Pre-Peritoneal (TAPP) approach was determined to be the best option for this patient so that bowel could be inspected and assessed for viability. Infraumbilical cut down and 3 port laparoscopy was performed demonstrating a 3.5 cm hernia defect in the left obturator canal. (Fig. 5) The sac was empty and the contents had reduced. There was a further 1 cm defect in the right obturator canal also with no contents. (Fig. 6). Small intestine was inspected from duodenal-jejunal flexure to terminal ileum and large bowel was inspected from caecum to rectum. All bowel was viable with a small area of congestion noted in the ileum – thought to be the now reduced contents of the hernia sac. The left sided peritoneal curtain was taken down above the obturator foramen to expose the contents and the hernia sac was dissected free from the surrounding fascia and structures. The defect was closed with continuous laparoscopic non-absorbable monofilament sutures and the peritoneal curtain re-hung with absorbable braided sutures. The very small right sided hernia sac was reduced from pre-peritoneal fascia and plicated to the anterior abdominal wall with absorbable braided laparoscopic sutures.

The patient returned to the ward and made an uneventful recovery. She was discharged from hospital on her second post-operative day with pain controlled and bowels opening. She was seen in Surgical Outpatient Clinic 4 weeks post operatively and has recovered well from the procedure. Her loin to groin pain has not recurred and she has returned to her normal active life.

Written consent was obtained from the patient prior to this report.

## 3 Discussion and review of literature

Following diagnosis, classical management of acutely incarcerated obturator hernia involved laparotomy. [[18], [19], [20]] As surgical techniques have evolved with time, a laparoscopic approach has been shown to be a safe alternative to laparotomy [[19], [20], [21]]. Furthermore the laparoscopic approach has shown to have faster recovery rates, reduced complications and reduced length of stay. (2, [19], [20], [21], [22])

**Fig. 1:**
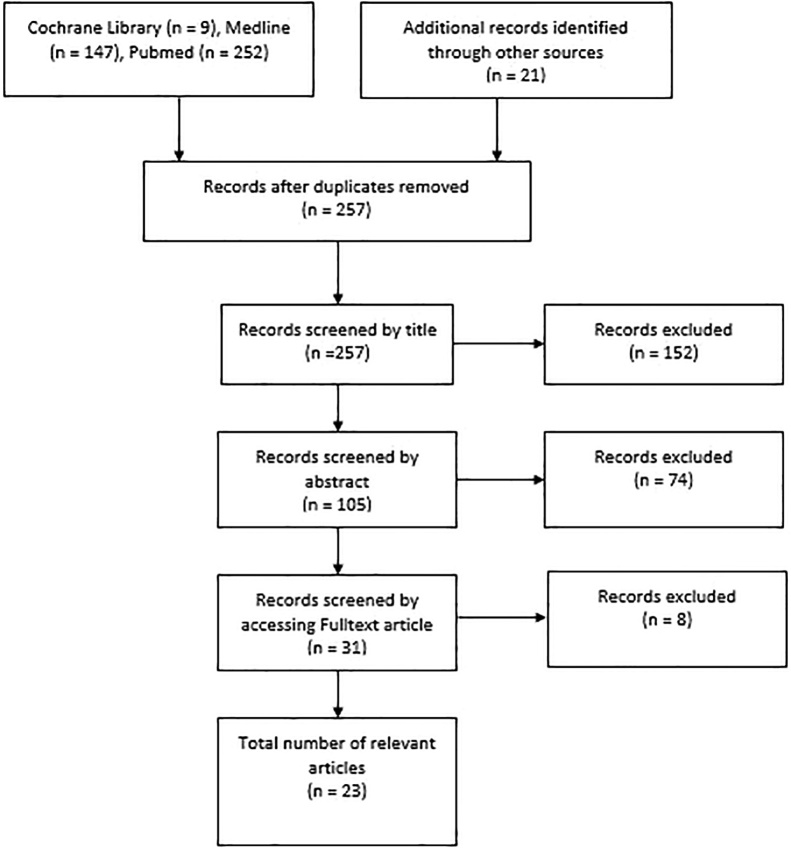
A comprehensive review of literature was conducted using Cochrane Library, Medline and Pubmed.

Initial searches of Cochrane Library, Medline and Pubmed yielded 273 results and after deletion of duplicates there were 257 publications. Filtration was initially conducted by title, followed by abstract review, followed by full text review. (Fig. 1). Most articles were case reports representing low level evidence. Of these articles 13 publications involved hip pain or musculoskeletal pain as a feature of presentation. [[23], [24], [25], [26], [27], [28], [29], [30], [31], [32], [33], [34], [35]] Two cases reported appendicitis within an obturator hernia; two reported bladder involvement of the hernia; one reported ureteric entrapment within the obturator foramen; one report of fallopian tube and one report of ovarian involvement. [[36], [37], [38], [39], [40]].A common feature of these reports were the diagnostic difficulty. Only one case prior to this involves ureteric colic as the presentation of obturator hernia – as reported by Izzo et al. [36]

**Fig. 2:**
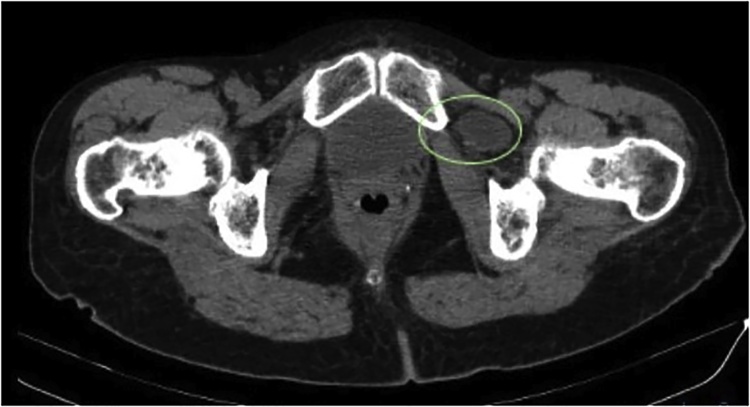
Axial CT imaging demonstrating small bowel within the obturator canal.

**Fig. 3:**
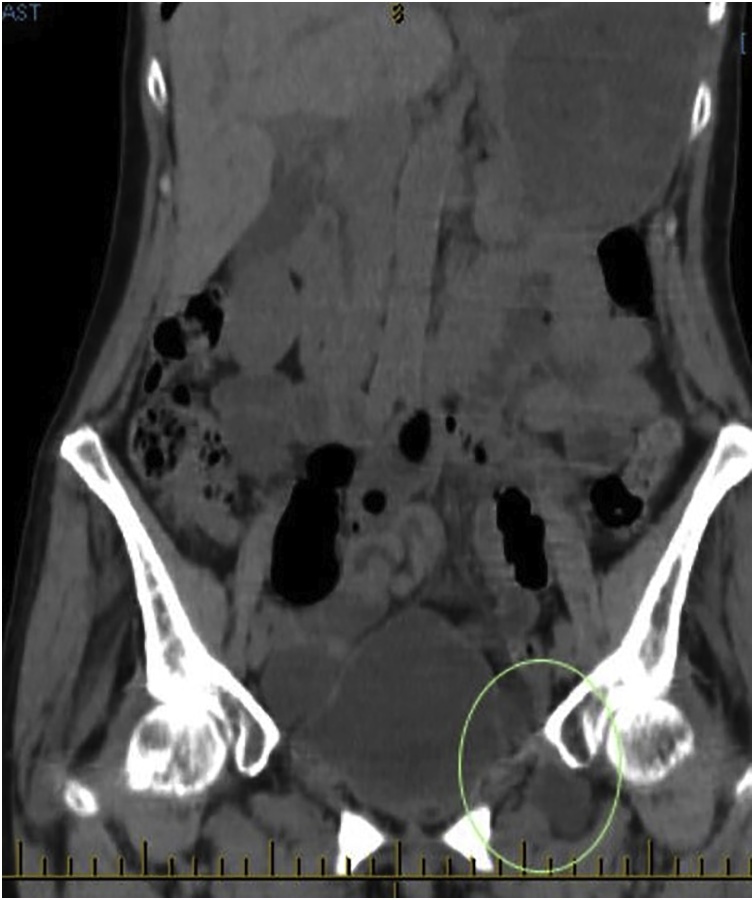
Coronal CT imaging demonstrating small bowel within the obturator canal.

**Fig. 4:**
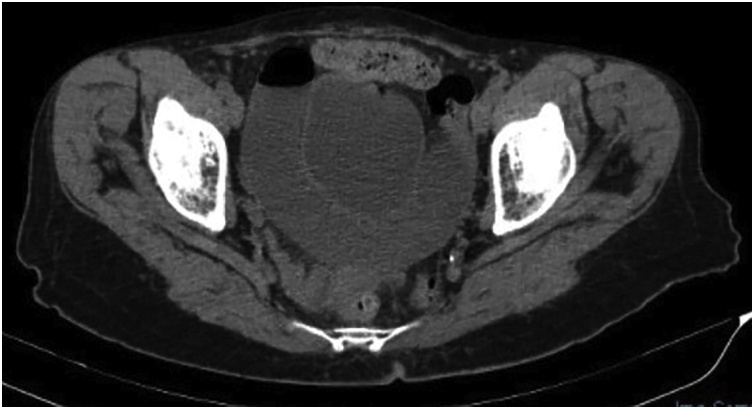
Axial CT imaging showing dilated small bowel loops proximal to the obturator foramen.

**Fig. 5:**
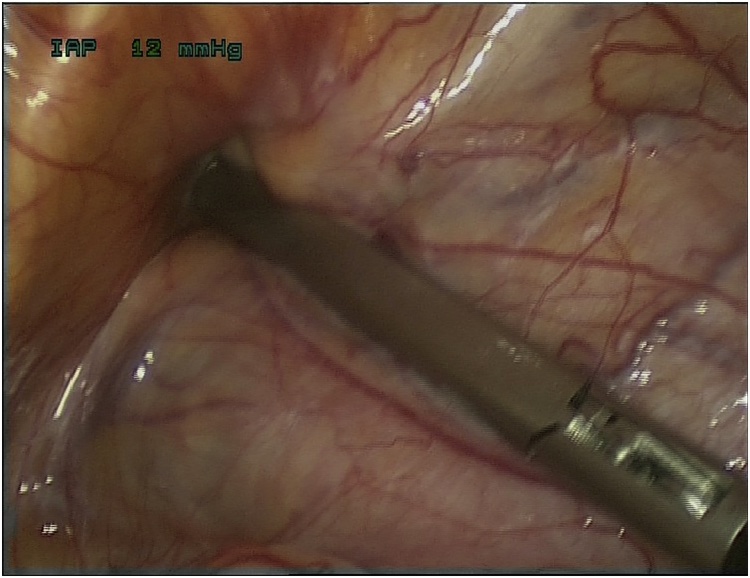
Intra-operative imaging of the hernia defect within the left obturator foramen.

**Fig. 6:**
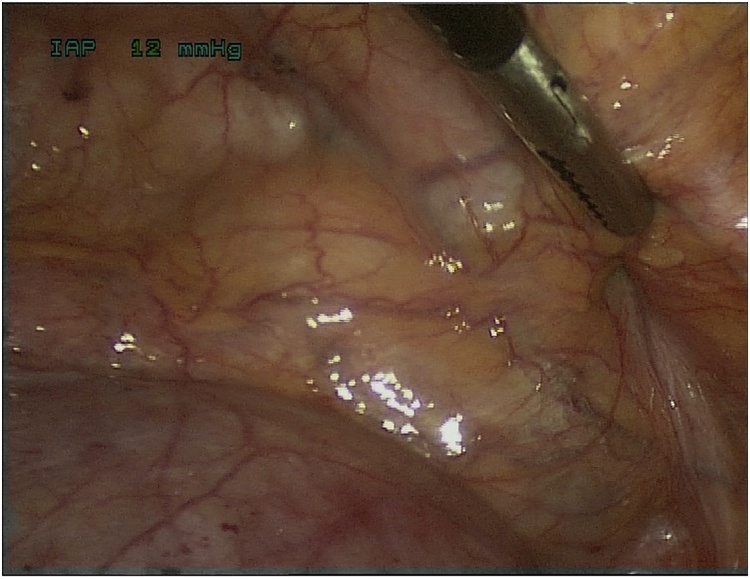
Intra-operative imaging showing a small hernia defect within the right obturator foramen.

The open approach via low laparotomy was the favoured surgical technique of choice for managing an acutely obstructed obturator hernia. With the development of laparoscopic equipment and techniques there is a growing body of evidence demonstrating superior patient outcomes with laparoscopic intervention. [[41], [42], [43], [44], [45], [46], [47]] Laparoscopic approaches – Trans Abdominal Pre Peritoneal (TAPP) and Total Extra Peritoneal (TEP) have demonstrated reduced length of stay, reduced post-operative pain and equivalent success in recurrence rates. [[41], [42], [43], [44], [45], [46], [47], [48], [49]].

Timing of CT imaging was crucial in this case. Obturator hernia is a diagnostic challenge and the transient behaviour of this patient’s disease further added to the dilemma. Fortunately, a hernia that spontaneously reduced did not cause significant morbidity to the patient, but the risk of future bowel infarction was certainly present. Scanning the patient whilst she was symptomatic made the diagnosis possible.

## 4 Conclusion

Obturator hernia is a rare condition that has unreliable clinical presentations. CT imaging early will improve likelihood of accurate diagnosis. Imaging investigations for transient symptoms have the highest yield when performed whilst the patient is symptomatic. Laparoscopic intervention is a safe and effective approach for emergency management of symptomatic obturator hernia.

## Consent

Written consent has been obtained from the patient involved in the case report.

## Author contribution

MH and HK developed the study concept together. The literature review was conducted by MH. The manuscript was originally written by MH and revised by HK.

## Registration of research studies

Not applicable – single case report

## Guarantor

Dr Matheesha Herath

## Provenance and peer review

Not commissioned, externally peer-reviewed

## Ethical approval

This manuscript is a single case report and is exempt from ethics review.

## Funding

This case report required no funding or sponsorship

## Declaration of Competing Interest

Nil conflicts of interest.
